# Drug diluent and efficacy of methylene blue in septic shock: authors’ reply

**DOI:** 10.1186/s13054-023-04633-0

**Published:** 2023-09-05

**Authors:** Miguel Ibarra-Estrada, Eduardo Kattan, Guadalupe Aguirre-Avalos, Glenn Hernández

**Affiliations:** 1 Unidad de Terapia Intensiva, Hospital Civil Fray Antonio Alcalde, Universidad de Guadalajara, Coronel Calderón 777, El Retiro, Guadalajara, Jalisco México; 2 Instituto Jalisciense de Cancerología, Guadalajara, Jalisco México; 3 The Latin American Intensive Care Network (LIVEN), Guadalajara, México; 4 Departamento de Medicina Intensiva, Facultad de Medicina, Pontificia Universidad Católica de Chile, Santiago, Chile

We thank Dubey et al. for their interest in our recently published randomized controlled trial (RCT) of the use of methylene blue (MB) in patients with septic shock [1]. They rise an interesting and justified debate about MB clinical administration.

We are aware that some manufacturers warn about possible precipitation of MB when diluted in 0.9% sodium chloride (normal saline, NS) [2]; however, this is not the case for all MB compounds, as the main clinical reference that supports this warning is an isolated report in which a hospital that usually diluted a generic formulation of MB in NS (as we do) had to switch to ProbayBlue® due to local shortage in 2017. During administration of the second dose, they found a precipitate in intravenous tubing with no harm to the patient [3].

Drug stability for infusions beyond 1–2 h could also be questioned due to scarcity of data, but MB has been safely diluted in NS in clinical studies even at higher concentrations than we used, including two other RCTs in patients with septic shock; our preparation consisted of a concentration of 0.2 mg/ml administered over 6 h, while Memis et al. used > 2 mg/ml over 6 h and Kirov et al. infused 5 mg/ml over 4 h [4, 5]. Based on these data and our previous unpublished local experience for a decade, we decided to use NS due to its more favorable distribution, as it is widely known that administration of 5% dextrose impacts mainly the intracellular compartment, with negligible effect on intravascular when compared to NS, even in cases when capillary integrity is present [6].

Drug manufacturers are usually compelled to warn about possible interactions of certain compounds based on pre-clinical data. A common example is norepinephrine bitartrate, whose manufacturers warn about possible inactivation if prepared with NS due to oxidation [7]; however, stability of norepinephrine in NS for prolonged time has been confirmed for decades [8, 9]. Patients are always closely monitored for any potential adverse effects in RCTs, and although our results could be considered as reassuring, we recognize the importance of post-marketing drug safety surveillance in real-world settings, and urge clinicians to strictly adhere to specifications of manufacturers along with local pharmacy department policies. Notwithstanding, we would like to pinpoint other pharmacokinetic/pharmacodynamic factors that could have improved the efficacy of MB in our trial even more than the use of NS; namely, an initial 1 mg/kg bolus [10], doses of 2–3 mg/kg instead of fixed 100 mg [11–13], continuous infusion [4, 14], repeated doses until shock resolution instead of a fixed 3-doses scheme, and especially, aiming for an earlier administration after septic shock diagnosis, as it has been suggested that efficacy could be enhanced if MB is used within the first 8 h [15].

## Data Availability

Not applicable.

## Abbreviations

MB: Methylene blue
NS: Normal saline
RCT: Randomized controlled trial
